# Oncogenic activation of FAK drives apoptosis suppression in a 3D-culture model of breast cancer initiation

**DOI:** 10.18632/oncotarget.11856

**Published:** 2016-09-06

**Authors:** Scott Walker, Fiona Foster, Amber Wood, Thomas Owens, Keith Brennan, Charles H. Streuli, Andrew P. Gilmore

**Affiliations:** ^1^ Wellcome Trust Centre for Cell-Matrix Research, Faculty of Biology, Medicine and Health, University of Manchester, Manchester, UK

**Keywords:** Focal adhesion kinase, breast cancer, apoptosis, anoikis

## Abstract

A key hallmark of cancer cells is the loss of positional control over growth and survival. Focal adhesion kinase (FAK) is a tyrosine kinase localised at sites of integrin-mediated cell adhesion to the extracellular matrix. FAK controls a number of adhesion-dependent cellular functions, including migration, proliferation and survival. Although FAK is overexpressed and activated in metastatic tumours, where it promotes invasion, it can also be elevated in cancers that have yet to become invasive. The contribution of FAK to the early stages of tumourigenesis is not known. We have examined the effect of activating FAK in non-transformed mammary epithelial cells (MECs) to understand its role in tumour initiation. In agreement with previous studies, we find FAK activation in 2D-culture promotes proliferation, migration, and epithelial-to-mesenchymal transition. However in 3D-cultures that better resemble normal tissue morphology, mammary cells largely respond to FAK activation *via* suppression of apoptosis, promoting aberrant acinar morphogenesis. This is an acquired function of FAK, because endogenous FAK signalling is not required for normal morphogenesis in 3D-culture or *in vivo*. Thus, FAK activation may facilitate tumour initiation by causing resistance to apoptosis. We suggest that aberrant FAK activation in breast epithelia is dependent upon the tissue context in which it occurs.

## INTRODUCTION

A hallmark of cancer is the loss of the normal positional control over cell proliferation, differentiation and apoptosis [1]. Focal adhesion kinase (FAK) is a non-receptor tyrosine kinase and adapter molecule that coordinates signalling pathways downstream of integrin-mediated adhesion to the extracellular matrix (ECM) [2]. FAK is recruited to integrin-based complexes in adherent cells, where autophosphorylation on tyrosine 397 (Tyr-397) allows FAK to form a complex with Src-family kinases. The subsequent Src-dependent phosphorylation of FAK on other tyrosine residues, along with sites on FAK-associated scaffold proteins such as p130CAS and paxillin, results in downstream signalling that regulates proliferation, differentiation and apoptosis. It is therefore not surprising that aberrant FAK signalling has been implicated cancer [3]. Consequently, there has been considerable interest in developing small molecule inhibitors of FAK.

FAK itself is not mutated in cancer. However, overexpression of FAK is often seen in solid tumours, which is associated with an increase in its activity as seen by phosphorylation on Tyr-397. FAK overexpression can occur through several mechanisms; for example amplification of the *FAK* gene or the loss of p53, which negatively regulates FAK expression [4–6]. Furthermore, increased FAK levels and activation often correlate with poor prognosis in invasive carcinomas [7, 8]. Several studies have examined the role of FAK in established mouse models of breast cancer, where it promotes tumour invasion and metastasis [9–12]. However, FAK overexpression is not restricted to invasive breast cancer, and is often seen in ductal carcinoma in situ (DCIS) [13]. FAK may therefore also contribute to the pre-invasive phenotype, although this possibility has not been explored.

In this study, we have examined the consequences of aberrant FAK activation in non-transformed mammary epithelial cells (MEC). Our data reveal that the effect of aberrant FAK activation is dependent upon cellular context. We find that activation of FAK in 2D-culture drives an EMT-like phenotype, increasing cell proliferation and migration. In contrast, FAK activation in 3D-culture results in the formation of aberrant acini *via* the suppression of apoptosis in those cells that are not in contact with the underlying basement membrane. Consequently, elevated FAK signalling is likely to have distinct roles at different stages of tumour development.

## RESULTS

### Constitutive FAK activation transforms normal mammary epithelial cells

**Figure 1:**
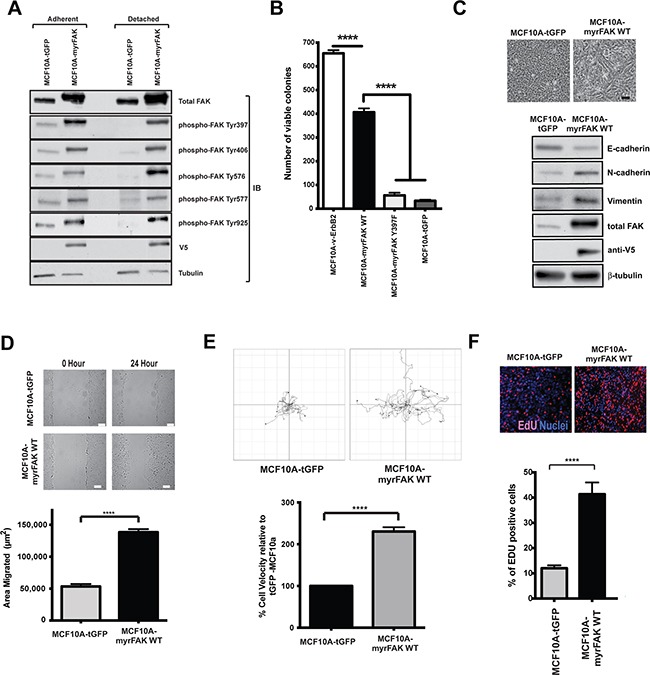
Constitutive activation of FAK in non-transformed MCF10A cells promotes colony formation in soft agar, EMT, migration and proliferation in 2D **A.** MCF10A mammary epithelial cells were stably infected with lentiviruses expressing either tGFP or myrFAK to mimic FAK overexpression and activation in breast cancer cells. To determine the level of FAK activation, lysates from both adherent and non-adherent cells were analysed by immunoblottting for total FAK, and FAK phosphorylation on tyrosines 397, 406, 576, 577 and 925. In tGFP expressing cells, all sites were phosphorylated on endogenous FAK in adherent cells, but lost following detachment. Phosphorylation on all sites was seen on myrFAK in both adherent and detached cells. Anti-V5 indicated the expressed myrFAK, and anti-tubulin was used as a loading control. **B.** MCF10A cells stably expressing v-ErbB2, myrFAK wildtype (WT), myrFAK Y397F or tGFP were plated as single cells in soft agar and grown for 7 weeks. Viable cells were stained with nitroblue tetrazolium and the number colonies quantified in three independent experiments. Data are the mean +/− SEM. Data were analysed by ANOVA. **** indicates p < 0.0001. **C.** Equal numbers of tGFP and myrFAK expressing MCF10A cells were cultured in 2D-monolayers. Images show confluent cultures. Scale bar = 25 μm. 24 hours post confluence, cells were lysed and analysed by immunoblotting with the indicated anti-bodies. **D.** Confluent 2D-monolayer cultures of tGFP and myrFAK MCF10A cells were scratch wounded, washed, and allowed to recover for 24 hours. Wound closure was quantified as the wound area occupied by cells after 24 hours. The data represent 15 fields of view from each of three independent experiments. Error bars = SEM. Significance was determined using student t-test. **** = p less than 0.0001. Scale bar = 150 μm. **E.** MCF10A cells expressing of tGFP or myrFAK cells were imaged every 15 minutes for 24 hours. Migration of individual cells were analysed using ImageJ. Shown are representative single cell migration tracks. For the graph, individual cell velocities were calculated and plotted relative to tGFP expressing cells. Data are the mean of >45 cells from three independent experiments. Error bars = SEM. Significance was determined using student t-test. **** = p less than 0.0001. **F.** Equal numbers of tGFP and myrFAK expressing MCF10A cells were pulse labelled with EdU for one hour prior to fixation, either 24 hours post plating. The percentage of EdU positive cells in each population was quantified from three independent experiments. Error bars = SEM. Significance was determined using ANOVA. **** = p less than 0.0001.

Several studies have shown that genetic deletion of FAK reduces the invasive potential and progression of established tumours [9–12, 14]. These findings are in keeping with work showing that FAK controls cell migration and focal adhesion turnover of cell lines in 2D-culture [15]. Given that FAK is often overexpressed and activated in pre-invasive breast tumours [13], we examined its role in the transformation of normal MECs. To investigate the role of FAK activation in pre-invasive breast cancer, we used an activated form of FAK (myrFAK), generated by attaching an N-terminal v-Src myristoylation sequence, which was also tagged at the C-terminus with a V5-epitope [16]. MCF10A cells were infected with pCDH-lentivirus expressing tGFP alone or myrFAK along with tGFP, and stably-expressing cells were selected by FACS. MCF10A-tGFP control cells showed normal adhesion dependent activation of endogenous FAK, seen by immunoblotting for the major phosphorylation sites (Figure 1A). In contrast, myrFAK remained phosphorylated on all of these sites in cells detached from the ECM (Figure 1A).

MCF10A cells stably expressing tGFP, myrFAK, myrFAK-Y397F (the Tyr-397 autophosphorylation site substituted to phenylalanine), or v-ErbB2 were seeded in soft agar and grown for seven weeks before determining the number of colonies (Figure 1B). Neither tGFP nor myrFAK-Y397F could support colony formation. However, myrFAK induced colony formation in soft agar, although not to the same extent as seen with v-ErbB2. Thus, FAK activation is sufficient to induce colony formation in a soft agar assay

In 2D-culture, MCF10A-tGFP cells form a regular monolayer with a cobblestone appearance (Figure 1C). In contrast, myrFAK cells, with higher levels of total FAK than controls, displayed a more spindle like morphology, along with reduced expression of E-cadherin, and increased N-cadherin and vimentin. The MCF10A-myrFAK cells migrated significantly faster than MCF10A-tGFP cells, both in a scratch wound assay (Figure 1D) and in single-cell time-lapse imaging (Figure 1E). Measuring DNA synthesis with EdU indicates that MCF10A-myrFAK cells cultured in 2D showed increased proliferation compared with MCF10A-tGFP cells (Figure 1F). Several studies have indicated that FAK promotes tumour cell invasion and migration [15], and our results reveal that MCF10A cells expressing myrFAK behave as expected in this regard.

To confirm that the observed phenotype of MCF10A cells was due to the activity of myrFAK, we treated cells with a small molecule inhibitor of FAK, AZ675. Proliferation and migration of MCF10A-myrFAK cells in 2D was inhibited by AZ675 (Supplementary Figure S1). AZ675 also blocked proliferation and migration in the control MCF10A-tGFP cells cultured in 2D, indicating that endogenous FAK is required for these behaviours in the parental cells (Supplementary Figure S1).

These results show that in 2D-culture, endogenous FAK promotes proliferation and migration. Moreover, its over-activation enhances this behaviour and drives a transition to an EMT-like state.

### Over expression of active FAK promotes MEC hyperplasia in 3D-culture

**Figure 2:**
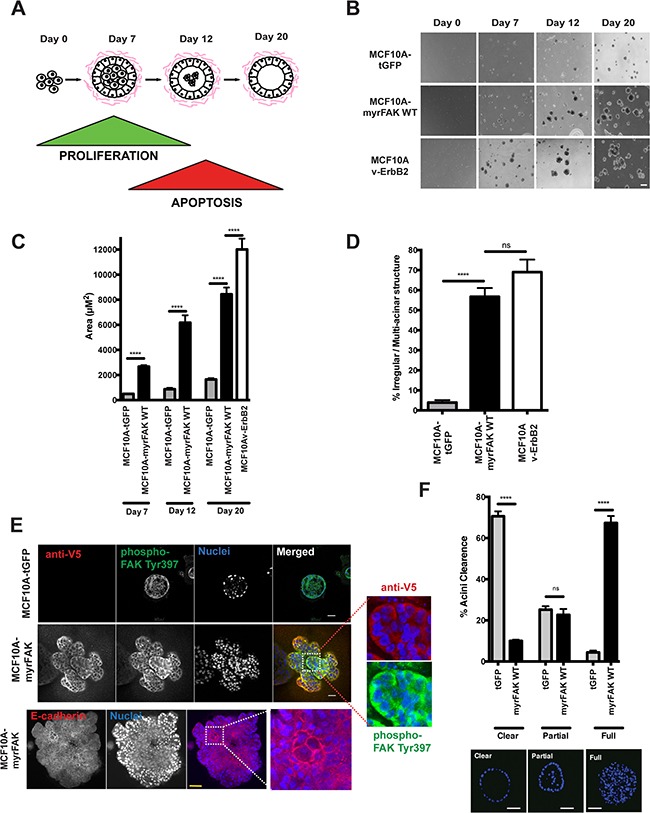
Constitutive activation of FAK results in aberrant acinar development in 3D-matrigel **A.** Schematic representation of MCF10A acinar morphogenesis in 3D-culture. Acini form over 20 days, due to an initial wave of proliferation followed by the central cells undergoing apoptosis. **B.** MCF10A cells expressing tGFP, myrFAK or v-ErbB2 were plated as single cell suspensions in matrigel for a total of 20 days. Representative images were taken on days 0, 7, 12 and 20. Scale bar = 100 μm. **C.** Quantification of acinar size from panel B. Acinar size was calculated using ImageJ, and represents the mean area of > 250 acini +/− SEM, from three independent experiments. **D.** The percentage of irregular/multilobular structures at Day 20 was calculated. Irregular structures were defined as having multiple lobules and/or a size over 4000 μm^2^. More the 250 structures per sample were examined, in three independent experiments. Error bars = SEM. Significance was determined using ANOVA. **** = p less than 0.0001. **E.** tGFP and myrFAK expressing MCF10A acini following 20 days culture in matrigel were fixed and immunostained for either anti-V5 and phospho-FAK Tyr397, or E-cadherin. Nuclei were stained with Hoechst. Equatorial confocal cross sections are shown. Scale bar = 25 μm. **F.** Acini from E. were quantified according to whether their lumens were clear of cells, partially filled or completely filled, independent of acinar size. Representative images of each classification are shown. Results represent mean of three independent experiments. Error bars =SEM. Data were analysed by ANOVA. **** = p < than 0.0001. Scale bar = 25 μm.

To determine the effect of aberrant FAK activation in a more physiological 3D-environment, cells were cultured in 3D-Matrigel. This is a well-established assay for mammary morphogenesis [17, 18]. In this model, MCF10A cells undergo proliferation to form cell clusters, followed by apoptosis of the central cells to form a hollow lumen [19] (Figure 2A). This process takes around twenty days. MCF10A cells deposit their own LM5-containing basement membrane around acini in Matrigel (Supplementary Figure S2).

Control MCF10A-tGFP cells form small acini, whereas MCF10A-vErbB2 cells grow into large, irregular structures (Figure 2B). MCF10A-myrFAK cells form intermediate acini that, whilst not normal in size, are not comparable to those expressing v-ErbB2. Quantifying both the acinar size (Figure 2C) and shape (Figure 2D) reveals that both myrFAK and v-ErbB2 alter normal morphogenesis. Confocal imaging of the 3D-acini shows that only the MCF10A-tGFP cells in contact with the ECM display FAK-Tyr-397 phosphorylation (Figure 2E). Nuclear staining shows that these acini also display hollow lumens (Figure 2F). In contrast, MCF10A-myrFAK acini are predominantly filled with cells. V5 immunostaining shows that myrFAK is expressed in every cell within the acini, with phosphorylated FAK-Tyr-397 present throughout. Furthermore, FAK phosphorylation is not restricted to the cell membrane, but is seen throughout the cell, unlike E-cadherin which is clearly membrane associated (Figure 2E). In contrast, in 2D-culture phosphorylated myrFAK is concentrated at sites of cell-ECM contact as well as throughout the cytoplasm (Supplementary Figure S2A). Phosphorylated FAK is excluded from nuclei, both in 2D and 3D. Despite the homogenous expression throughout the acini, the distribution of both α6-integrin and LM5 shows that myrFAK has no effect on the formation of a LM5-rich basement membrane on the outside of the structure (Supplementary Figure S2B and S2C). MCF10A-myrFAK cells in 3D culture maintain E-cadherin mediated junctions throughout the acini.

These results show that in 3D-culture, aberrant FAK activation disrupts normal acinar morphogenesis by causing lumen filling. However, it does not impede the formation of a correctly positioned basement membrane.

### The effect of aberrant FAK activation in 3D-culture is distinct to that seen in 2D

**Figure 3:**
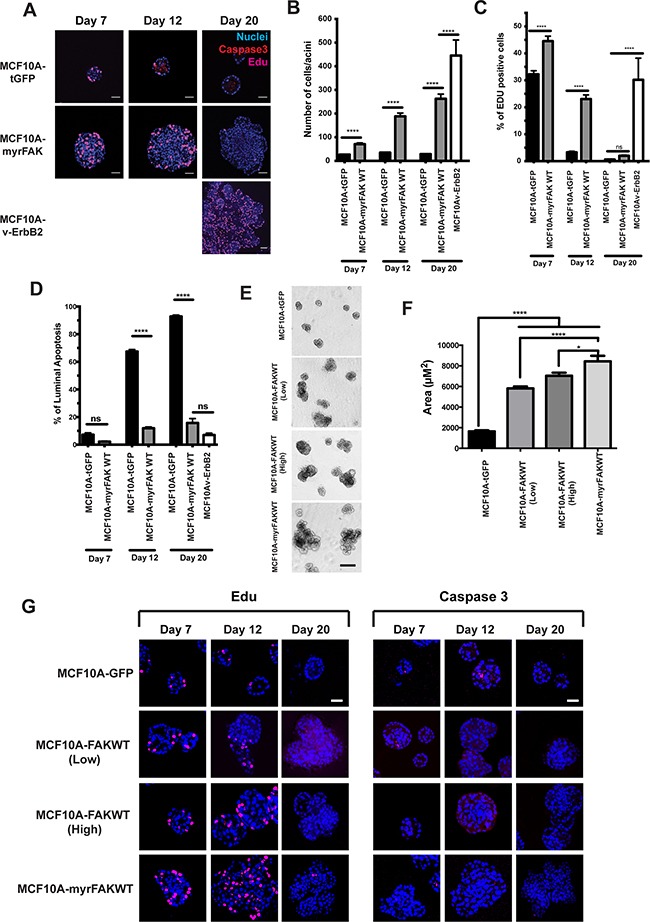
FAK suppresses apoptosis in 3D acini, but does not drive constitutive proliferation **A.** MCF10A cells stably expressing tGFP, myrFAK or v-ErbB2 were cultured in matrigel for up to 20 days. Cells were pulse labelled for one hour with EdU prior to fixation. Cells were immunostained for activated caspase 3 and EdU, and nuclei stained with Hoechst. Representative equatorial confocal cross sections are shown. Scale bar = 25 μm. **B.** Quantification of the number of cells in each equatorial cross section. Each time point represents ~ 100 acini from three independent experiments. Error bars =SEM. Data were analysed by ANOVA. **** = p < than 0.0001. **C.** Quantification of the percentage of EdU positive cells per acini. Data are from ~ 100 acini from three independent experiments. Error bar are SEM. Data were analysed by ANOVA. **** = p < than 0.0001. **D.** Quantification of the percentage of luminal cells that stain positive for activated caspase 3. Cells in the outer layer of the acini, and which would be in direct contact with the ECM, were not positive for caspase 3 and are not included in this analysis. This is particularly apparent in the tGFP expressing cells at day 12 and day 20 shown in A. Data were analysed by ANOVA. **** represents p < 0.0001. **E.** MCF10A cells stably expressing tGFP, myrFAK or non-myrstiolated FAK (FAKWT, both low and high expressing populations selected by FACS) were cultured in matrigel for 20 days. Representative images taken on day 20. Scale bar = 100 μm. **F.** Quantification of acinar size from **E.** Acinar size was calculated using ImageJ, and represents the mean +/− SEM, from three independent experiments. **G.** MCF10A cells stably expressing tGFP, myrFAK and FAKWT were cultured in matrigel for up to 20 days. Cells were pulse labelled for one hour with EdU prior to fixation. Cells were immunostained for EdU (left panel) activated caspase 3 (right panel), and nuclei stained with Hoechst. Representative equatorial confocal cross sections are shown. Scale bar = 25 μm.

MCF10A cells expressing tGFP, myrFAK or v-ErbB2 were cultured in 3D-Matrigel, and examined for proliferation and apoptosis (Figure 3A). As expected, both MCF10A-myrFAK and MCF10A-v-ErbB2 acini contained significantly more cells than control MCF10A-tGFP (Figure 3B). However EdU labelling revealed that, unlike v-ErbB2, FAK activation does not drive un-constrained proliferation (Figures 3A and 3C). Although MCF10A-myrFAK cells show more proliferation than MCF10A-tGFP cells in early cultures, by day 20 proliferation drops to basal levels. In contrast, MCF10A-v-ErbB2 cells retain sustained proliferation. Thus, aberrant FAK activation does not drive unrestricted proliferation in a 3D-context.

We also examined apoptosis in 3D-cultures of MCF10A cells (Figures 3A and 3D). tGFP expressing cells show an onset of apoptosis at day 12, which selectively occurs in those cells in the centre of the acini and not in direct contact with the ECM (Figure 3A). In contrast, there is an almost complete absence of apoptosis throughout MCF10A-myrFAK acini. v-ErbB2 also potently supresses luminal apoptosis. Thus, there is a major difference in apoptosis between MCF10A-tGFP and MCF10A-myrFAK cells.

We asked if overexpression of non-myristioylated FAK leads to a similar phenotype to myrFAK. To do this, we generated MCF10A cells over-expressing wildtype FAK (FAK-WT) without the N-terminal myr-Tag, sorted these into two populations by FACS according to “high” and “low” tGFP expression, and compared them with cells expressing tGFP and myrFAK (Figure 3E). Although FAK-WT overexpression did not result in acini of the same size as those expressing myrFAK, they are larger than tGFP cells (Figure 3F). We examined proliferation and apoptosis by staining with EdU and active caspase 3 (Figure 3G). Overexpressing FAK-WT recapitulates the effect of expressing myrFAK, because at day 12 there is more proliferation in the “high” population. In contrast, the tGFP controls show luminal apoptosis, which is suppressed in both “high” and “low” FAK-WT expressing cells.

**Figure 4:**
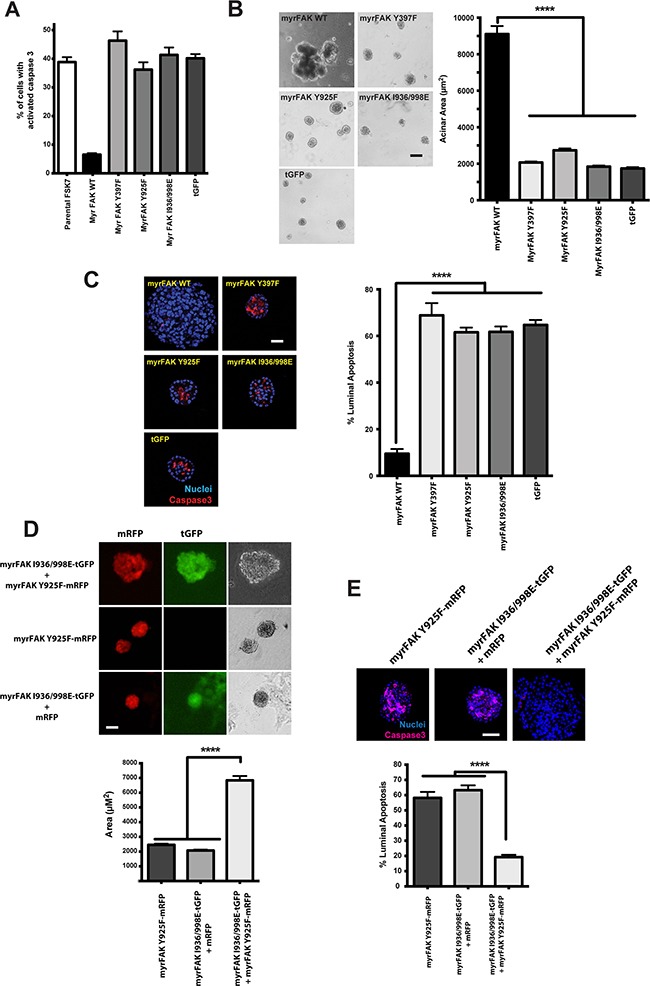
myrFAK suppression of luminal apoptosis requires tyrosine 925 phosphorylation and the paxillin binding site **A.** myrFAK supresses anoikis in FSK-7 mouse mammary epithelial cells. Uninfected FSK-7 cells, and those stably expressing tGFP, myrFAK wildtype (WT), myrFAK-Y397F, myrFAK-Y925F, myrFAK-I936/998E (paxillin binding defective), were detached from ECM and maintained in suspension for 24 hours. Apoptosis was quantified by active caspase 3 immunostaining. Data represent the mean of three independent experiments. Error bars = SEM. **B.** MCF10A cells stably expressing tGFP, myrFAK-WT, myrFAK-Y397F, myrFAK-Y925F or myrFAK-I936/998E were seeded as single cells in matrigel and cultured for a total of 20 days. Representative bright field images at day 20 are shown, and acinar area was quantified using ImageJ. Data represent the means from three independent experiments. Error bars =SEM. Data analysed by ANOVA. **** = p<0.0001. Scale bar = 50 μm. **C.** MCF10A cells stably expressing tGFP, myrFAK-WT, myrFAK-Y397F, myrFAK-Y925F or myrFAK-I936/998E were cultured in 3D-matrigel for 10 days, then fixed and immunostained for active caspase 3. Nuclei were stained with Hoechst. Equatorial confocal images were captured, and apoptosis of luminal cells quantified as a percentage of the total luminal cell population. Representative images are shown. Data represent the means from three independent experiments. Error bars = SEM. Data were analysed by ANOVA. **** = p < 0.0001. Scale bar = 25 μm. **D.** MCF10A cells were stably infected with: pCDH-myrFAKI936/998E/tGFP plus pCDH-mRFP; pCDH-myrFAK-Y925F/mRFP; or pCDH-my.rFAKI936/998E/tGFP plus pCDH-myrFAK-Y925F/mRFP. Cells were grown in 3D-matrgel for 20 days, and imaged for mRFP/tGFP. Representative bright field images at day 20 were quantified using ImageJ. Data represent the means from three independent experiments. Error bars =SEM. Data analysed by ANOVA. **** = p<0.0001. Scale bar = 50 μm. **E.** Cells from D. were cultured in 3D-matrigel for 10 days before quantifying apoptosis by immunostaining for active caspase 3. Data represent the means from three independent experiments. Error bars = SEM. Data were analysed by ANOVA. **** = p < 0.0001. Scale bar = 25 μm.

We previously found that anoikis of MECs is supressed by myrFAK, but not by non-functional or partially-functional myrFAK lacking specific docking sites for downstream effectors [16]. Substitution of the major auto-phosphorylation (Y397F), the Grb2 binding (Y925F) or the paxillin-binding (I936/998E) sites each prevent anoikis suppression (Figure 4A). Similarly, none of these myrFAK variants supported the formation of aberrant MCF10A acini (Figure 4B), and they show luminal apoptosis that is comparable to that seen in MCF10A-tGFP acini (Figure 4C).

This result suggests that two independent pathways downstream of FAK, activated *via* Tyr-925 and paxillin, are required for the ability of FAK to suppress luminal apoptosis. To test this, we generated two pCDH-lentivirus, one expressing myrFAK-Y925F with mRFP, and the other myrFAK I936/998E with tGFP (Figure 4D). MCF10A cells expressing these constructs individually form normal acini with comparable levels of luminal apoptosis (Figures 4D and 4E). In contrast, co-expressing both myrFAK variants results in large acini and apoptosis suppression. Thus, each partially-functional FAK was able to complement the lost function of the other.

These results show that the effect of aberrant FAK activation in 3D-culture occurs principally *via* suppression of apoptosis in cells that are not in contact with the ECM. Moreover, two parallel signalling pathways downstream of FAK are needed to regulate this apoptosis suppression. This phenotype is distinct from that seen in 2D-culture, revealing that the context in which FAK signalling occurs is important with regard to the outcome.

### FAK activity is required to maintain aberrant acinar morphology

**Figure 5:**
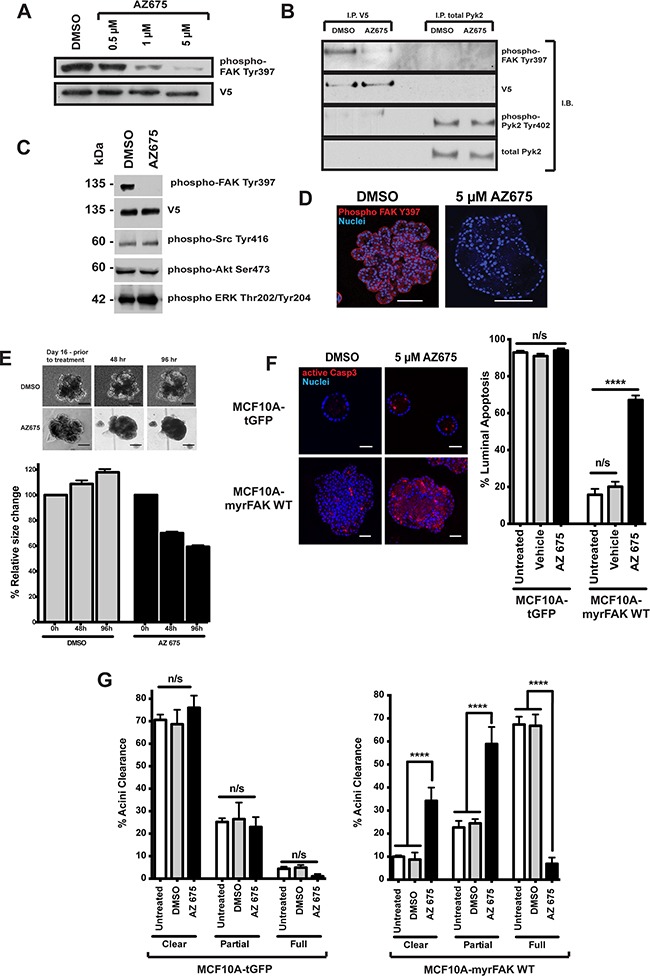
Inhibition of FAK in 3D-mammary cultures selectively induces apoptosis in the luminal cells **A.** MCF10A-myrFAKWT expressing cells grown in 2D were treated with DMSO or the FAK inhibitor, AZ675, a novel small molecule ATP analogue, at the indicated concentrations. Cell lysates were then immunoblotted for FAK Tyr-397 phosphorylation (the autophosphorylation site to determine FAK kinase activity), and V5. **B.** MCF10A-myrFAKWT expressing cells were treated with DMSO or 5 μM AZ675. myrFAK was immunoprecipitated (I.P) using anti-V5, and endogenous Pyk2 with anti-Pyk2. Immunoprecipates were then immunoblotted (I.B.) with the indicated antibodies. AZ675 did not inhibit phosphorylation of Pyk2 on Tyr-402. **C.** myrFAK expressing MCF10A cells were treated with 5μM AZ675 or DMSO for 24 hours in 2D culture. Lysates were analysed by immunoblotting with the indicated antibodies. FAK Tyr397 phosphorylation was abolished, but there was no effect on Src, Akt or Erk phosphorylation on their key regulatory sites. **D.** myrFAK expressing MCF10A cells were grown in matrigel for 16 days, and then treated with either DMSO or AZ675 for a further 96 hours before fixing and immunostaining for phospho-FAK Tyr397. AZ675 abolished FAK phosphorylation throughout the acini, which appeared to develop cleared regions within the lumen. Scale bar = 50 μM. **E.** myrFAK acini were grown for 16 days, before being treated with either DMSO or AZ675 for 96 hours. Images were taken before drug addition, and then at 48 and 96 hours. The images show the same acini at each time point. Scale bar = 50 μm. Acinar area was measured for ~ 50 acini per condition at the three timepoints, using FIJI. The data are plotted as the relative change in individual acini area relative to day 16, prior to treatment. Error bars = SEM. Scale bar = 50 μM. **F.** tGFP and myrFAK MCF10A cells were grown in marigel for 16 days, and then treated with DMSO or AZ675 for 96 hours. Cells were then fixed and immunostained for active caspase 3. Equatorial confocal sections were captured, and the percentage of caspase 3 positive luminal cells was quantified as in Figure 4. Data represent the mean of three independent experiments, with > 100 acini. Error bars = SEM. Data were analysed by ANOVA. Scale bar = 25 μM. **G.** The tGFP and myrFAK acini from D. were analysed for acinar clearance, using the criteria from Figure 2. Data represent the mean of three experiments with > 100 acini. Error bars = SEM. Data were analysed by ANOVA.

To determine if sustained FAK activity is required to maintain the aberrant morphology of MCF10A-myrFAK acini, we treated established acini with the novel FAK inhibitor AZ675. A dose response shows that 5 μM AZ675 inhibited the majority of FAK Tyr-397 phosphorylation (Figure 5A), but has no effect on phosphorylation of the FAK homologue, Pyk2 (Figure 5B). 5 μM AZ675 also does not alter phosphorylation of Src, Akt or Erk (Figure 5C).

MCF10A-myrFAK grown for 16 days and treated for a further 96 hours with 5μM AZ675 show complete loss of FAK Tyr-397 phosphorylation throughout the acini, which is not seen in DMSO treated MCF10A-myrFAK acini (Figure 5D). Furthermore, AZ675 treated MCF10A-myrFAK acini progressively shrank and lost their multi-lobular shape (Figure 5E).

AZ675 has no significant effect on apoptosis in MCF10A-tGFP acini, shown by active caspase 3 staining, but causes significant cell death throughout the core of MCF10A-myrFAK acini (Figures 5F, S3A). However, it does not induce apoptosis in MCF10A-tGFP or MCF10A-myrFAK cells that are in contact with the ECM. AZ675 also has no effect on proliferation in these acini (Supplementary Figure S3B), or on the number of cleared, partially cleared, or full acini formed by MCF10A-tGFP cells (Figure 5G). In contrast, inhibiting FAK with AZ675 induced a significant reversal of the aberrant morphology of MCF10A-myrFAK acini (Figure 5G).

**Figure 6:**
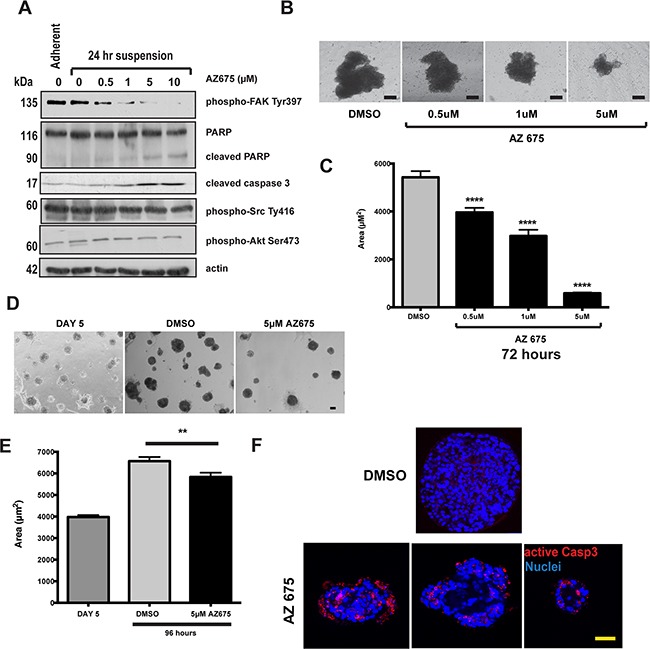
4T1 breast cancer cells that show adhesion independent FAK signalling are sensitive to FAK inhibition **A.** 4T1 cells were grown either attached or in suspension for 72 hours, and then treated with the indicated concentrations of AZ675 for 24 hours. Cells were lysed in RIPA buffer and immunoblotted using the indicated antibodies. As in MCF10A cells, AZ675 did not inhibit Src or Akt phosphorylation. **B.** Equal numbers of 4T1 cells were grown in suspension in the presence of either DMSO or the indicated concentrations of AZ675 for 72 hours. Representative images of spheroids formed at the end of the time period are shown. Scale bar = 25 μm. **C.** Quantification of the area of 4T1 spheroids from the experiment in B. Data represent > 500 spheroids from three independent experiments. Error bas = SEM. Data were analysed by ANOVA. **** = p< 0.0001. **D.** 4T1 cells were plated as single cells in matrigel and cultured for 5 days. At day 5, cells were fed with fresh culture media containing either DMSO or 5 μM AZ675, and cultured for a further 96 hours. Representative images are shown at day 5, prior to treatment, and following treatment for 96 hours. Scale bar = 50 μm. **E.** Quantification of acinar area from panel D. Acinar size was measured using ImageJ, and represent the mean of > 250 acinar structures from three independent experiments. Error bars = SEM. Data were analysed by ANOVA. ** = p < 0.05. **F.** 4T1 cells in 3D-matrigel following 5-day pre-treatment and 96 hours in DMSO or 5 μM AZ675. Cells were immunostained for active caspase 3. Three representative images of AZ675 acini are shown, indicating that, unlike in MCF10A cells, apoptosis in 4T1 cells is not spatially restricted to the luminal cells. Scale bar = 50 μm.

To confirm the results obtained with AZ675 with another cell line, we examined its effect in 4T1 mouse mammary cancer cells. These cells undergo FAK-dependent metastasis in an orthotopic mouse model [20], and inhibiting FAK induces apoptosis in them [21]. 4T1 cells show levels of FAK activation after 24 hours in suspension that are equivalent to that in adherent cells, indicating that FAK is aberrantly activated in these like MCF10A-myrFAK cells (Figure 6A). AZ675 treatment inhibits FAK phosphorylation, activates capase 3, and induces cleavage of PARP, but has no effect on Src or Akt phosphorylation in suspension-cultured cells (Figure 6A). 4T1 cells grow as clusters in suspension, and these are significantly reduced in size by AZ675 treatment (Figure 6B, 6C). In 3D-Matrigel cultures, 4T1 cells grow into large, irregular shaped acini similar to those seen with MCF10A-myrFAK (Figure 6D,E). Again, the growth of these acini is significantly inhibited by AZ675 treatment. However, unlike MCF10A-myrFAK cells, FAK inhibition results in apoptosis throughout the 4T1 acini, and is not restricted just to cells within the lumen (Figure 6F).

These results show that blocking FAK kinase activity with AZ675 is specific for FAK, and that this blocks the cell-filled phenotype of aberrant acini in myrFAK expressing MCF10A.

### FAK inhibition does not affect normal acinar development

**Figure 7:**
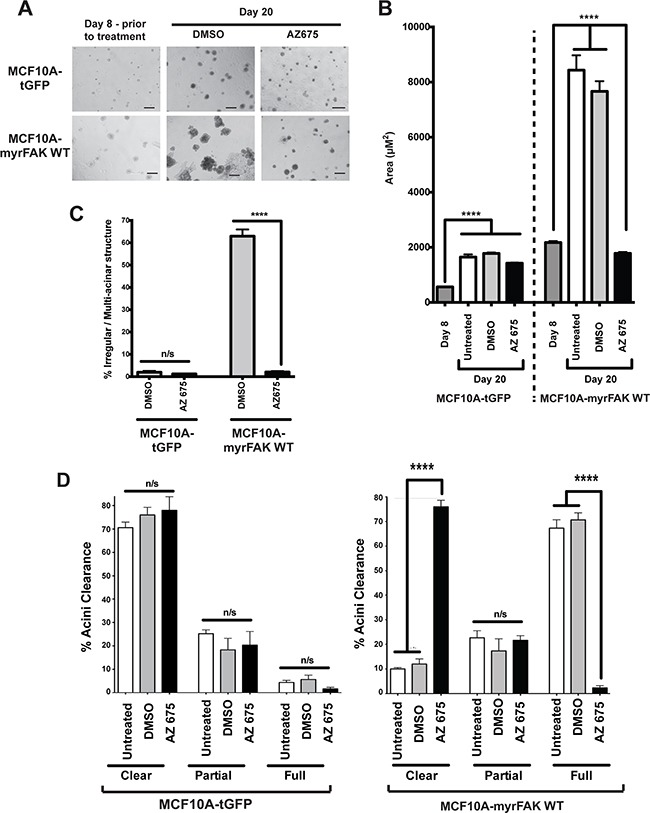
Normal acinar morphogenesis in 3D cultures does not require endogenous FAK signalling **A.** tGFP or myrFAK MCF10A cells were plated as single cells in matrigel for 8 days, and then treated with either DMSO or 5 μM AZ675 for a further 12 days, feeding cells with fresh media and drugs every 4 days. Representative brightfield images are shown. Scale bar = 100 μm. **B.** The mean acinar size of tGFP or myrFAK MCF10A was quantified in 3D-matrigel at day 8 prior to treatment and then after a further 12 days either untreated, or with either DMSO or 5 μM AZ675 (total of 20 days). > 250 acini were analysed from three independent experiments. Error bars = SEM. Data were analysed by ANOVA. **C.** The number of irregular shaped acini from A. was quantified at Day 20, following treatment with either DMSO or AZ675. **D.** tGFP and myrFAKWT expressing MCF10A cells were cultured in matrigel for 8 days and then treated with DMSO for 12 days as in A. Cells were fixed and stained with Hoechst, and acinar clearance quantified from equatorial confocal sections as in Figure 3. Data represent the mean from three independent experiments. Error bars = SEM. Data were analysed by ANOVA.

As we observed no apoptosis in those cells directly contacting the ECM following FAK inhibition, we asked if inhibiting FAK earlier would affect the development of hollow acini. Cells were cultured in 3D-Matrigel for eight days, then AZ675 was added for the remaining time up to day twenty, replenished every four days. Inhibiting endogenous FAK had no effect on the formation of MCF10A-tGFP acini (Figure 7). However, AZ675 prevented the irregular development of MCF10A-myrFAK acini, which now formed acini that were indistinguishable in size and morphology from the MCF10A-tGFP controls.

To further test whether FAK has a role in normal mammary development, we compared the glands of WT and *FAK fx/fx;BLG-Cre Tg* mice that had been put through two rounds of pregnancy. In these mice, LoxP sites flank both copies of the FAK gene to enable its deletion by Cre recombinase [22]. The endogenous FAK gene is deleted during the first pregnancy when Cre is expressed under the β-lactoglobulin (BLG) promoter, allowing its role in acinar formation to be examined during the second pregnancy. Note that although FAK deletion results in compensatory up regulation of Pyk2 in mouse embryonic fibroblasts, which can be reversed by expression of exogenous FAK (Supplementary Figure S4A), there was no effect of its deletion on Pyk2 levels in the mammary gland *in vivo* (Supplementary Figure S4B).

**Figure 8:**
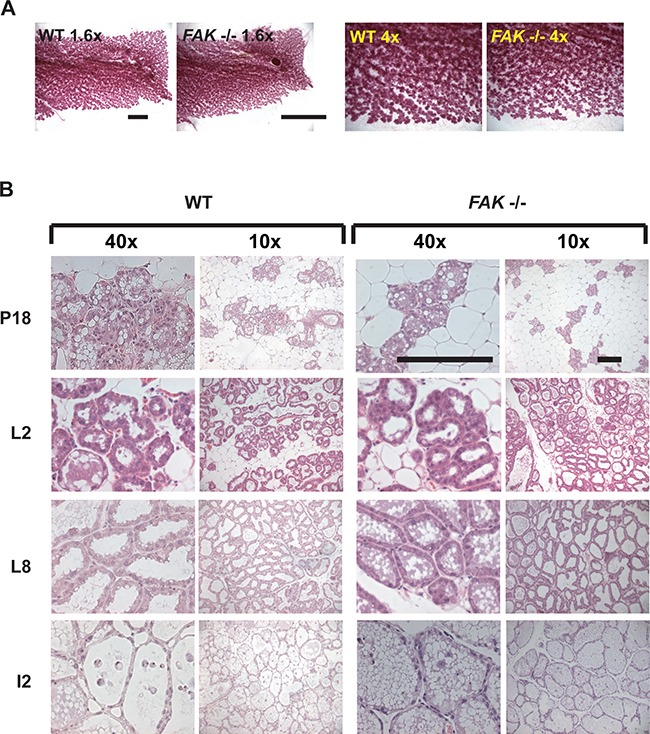
FAK is not required for normal mammary gland function *in vivo* **A.** Wildtype (WT) and *FAK fx/fx;BLG-Cre Tg* (*FAK* −/−) mice were put through two rounds of pregnancy, and mammary glands isolated on day 18 (P18) of the second pregnancy. Whole mounts of the glands were prepared and show no significant difference in the presence of absence of FAK. Scale bar = 5 mm. **B.** H and E staining of mammary glands from second pregnancy P18, lactation day 2 and 8 (L2, L8), and involution day 2 (I2) wildtype (WT) and *FAK fx/fx;BLG-Cre Tg* (*FAK* −/−) mice. There was no difference in the morphology of the acini, and both showed the clear presence of fat globules indicative of milk production. Scale bar = 800 μM.

There was no difference in the ability of the WT or *FAK fx/fx;BLG-Cre Tg* mice to suckle pups, and indeed the extent to which the mammary tissue developed and filled the fat pad was identical in WT and FAK deficient glands (Figure 8A). Moreover in a second pregnancy, the acini present in late pregnancy, lactation and early involution show no differences between WT and *FAK fx/fx;BLG-Cre Tg* mammary glands (Figure 8B).

These results show that FAK is not required for the formation of functional acini, either in 3D-culture or *in vivo*. However, our results reveal that the aberrant activation of FAK causes changes in MEC function that are similar to those seen in the early stages of cancer.

## DISCUSSION

The data presented here reveal that the function of FAK in non-transformed epithelia is highly dependent upon cellular context. In 2D-culture, FAK is required for proliferation and migration, and its aberrant activation drives EMT-like changes in non-transformed MECs. By contrast in 3D-culture or *in vivo*, endogenous FAK is not required for normal MEC morphogenesis or function. However, constitutive FAK activation in normal cells results in aberrant acinar morphogenesis in 3D-culture, through the suppression of apoptosis in cells within the central lumen. Our results reveal that inappropriate activation of FAK allows it to acquire functions that are not associated with normal epithelial biology.

Several studies have indicated that FAK has a role in cancer progression, promoting interest in developing FAK inhibitors [21, 23–25]. Many of these studies have used *in vivo* models where another oncogene drives tumorigenesis, and endogenous FAK has been genetically deleted in those tumours. For example, MMTV-PyMT induces mouse mammary tumours that quickly become invasive; however in these cancers FAK deletion increases latency and reduces invasion [10]. Similar results have been obtained in an oncogenic ErbB2 tumour model [9]. In both the MMTV-PyMT and v-ErbB2 models, the loss of FAK does not prevent tumour onset or eventual progression. However, these studies have not addressed the role of aberrant FAK signalling in pre-invasive tumours, or how FAK activation contributes to tumour initiation.

FAK is often aberrantly activated in pre-invasive mammary DCIS [13]. Normal mammary morphogenesis can be modelled using 3D-cultures, in which non-transformed MCF10A cells form polarised, growth-arrested, acini that are surrounded by a basement membrane. The acini develop hollow lumens, and form through proliferation followed by apoptosis of those cells within centre of the structure. The phenotype of MCF10A cells expressing activated FAK in 3D is reminiscent of DCIS, where epithelial cells develop a perturbed architecture and occupy the lumen, but fail to disrupt the surrounding basement membrane [26]. This may be explained by the ability of aberrant FAK signalling to overcome the suppressive environment of normal epithelium, a phenotype seen with other oncogenes [27]. Perturbation of this normal morphogenesis may result from increased proliferation, decreased apoptosis, or altered positional regulation of these processes.

A key driver of the aberrant morphology of MCF10A-myrFAK acini is the suppression of apoptosis within the centre of the acinus [19]. The importance of apoptosis regulation by FAK in cancer is not clear, as it is often studied in the context of other oncogenes that potently suppress apoptosis. Although active FAK can prevent anoikis in detached cells, it is probably not needed to provide a survival signal in adherent cells [16]. Indeed, inhibiting endogenous FAK or myrFAK in 2D-cultures of MCF10A cells does not induce apoptosis. Furthermore, deleting of FAK *in vivo* does not produce an overt mammary cell death phenotype. In contrast though, inappropriate FAK activation allows MECs to survive away from the ECM. In 3D-culture, MECs on the periphery of the acini obtain a FAK-independent survival signal from the ECM. myrFAK results in luminal cells acquiring apoptosis resistance, allowing them to survive away from their normal positional context. Interestingly when myrFAK was inhibited causing the acini to take on a normal morphology, only the luminal cells undergo apoptosis with the peripheral cells surviving.

FAK signalling also regulates proliferation, and it enhances the proliferative potential of oncogenic ErbB2 *in vivo* [9, 28]. Indeed, our unpublished data suggest that myrFAK enhances v-ErbB2 tumour growth in MCF10A xenografts, although myrFAK alone does not drive tumour formation (Foster, Walker and Gilmore, unpublished data). However, the role of FAK in cell proliferation is context dependent. In 2D-culture, endogenous FAK contributes to proliferation, and myrFAK enhances this. AZ675 inhibits proliferation in both WT and myrFAK expressing MCF10A cells in 2D-culture. However the role of FAK in proliferation in 3D-culture is significantly different from that in 2D. A mature epithelial microenvironment provides a suppressive influence on cells, preventing growth into the lumen [27]. Inhibiting endogenous FAK did not alter normal acinar formation in 3D-culture, or even mammary gland development *in vivo*. In contrast myrFAK expression liberates MECs from positional suppression, allowing proliferation of cells within the lumen of developing acini. However, it does not drive the unconstrained proliferation seen in MECs expressing v-ErbB2. myrFAK cells show a prolonged window of proliferation, but ultimately become growth arrested, even though acini failed to hollow. Thus, inappropriate FAK activation in 3D allows it to acquire functions that endogenous FAK does not provide.

How FAK signals in different contexts is linked to its complex scaffolding interactions and the subcellular locations in which these occur [29–31]. FAK signals both within and beyond the adhesome, with specific functions associated with its recruitment to distinct subcellular compartments and association with different downstream signalling partners. FAKs ability to supress anoikis has been linked with both nuclear and cytoplasmic functions, and recently with integrin complexes on endosomes [29, 30]. myrFAK is recruited to focal adhesions in MCF10A cells cultured in 2D, but also found in the cytosol. However in 3D, myrFAK was not obviously concentrated at the cell membrane. Our data indicate that different FAK complexes signalling independently, through paxillin and Tyr-925, jointly contribute to anoikis suppression and acinar hyperplasia. It will be intriguing to determine if these complexes exist within different subcellular compartments.

In summary, our data reveal that the consequences of FAK signalling depend upon the microenvironmental context of the cell. Whereas endogenous FAK does not play a significant role in either the survival or proliferation of non-transformed MEC in 3D-culture or *in vivo*, aberrant FAK signalling is sufficient to drive a pre-invasive phenotype in this context. In future, a possibility that needs to be explored is that overexpression of FAK allows it to acquire new functions that contribute to pre-invasive disease.

## MATERIALS AND METHODS

### Cell culture

MCF10A mammary epithelial cells (MECs) were cultured in DMEM-F12 supplemented with 5% horse serum, 100 ng/ml cholera toxin, 20 ng/ml EGF, 0.5 μg/ml Hydrocortisone, 10 μg/ml insulin. 4T1 mouse mammary cancer cells [32] were cultured in RPMI supplemented with 10% foetal bovine serum. Human embryonic kidney 293T cells were cultured in DMEM with 10% foetal bovine serum. FSK-7 MECs (Kittrell et al., 1992) were cultured as previously described (Gilmore et al., 2000).

### Antibodies and inhibitors

The antibodies and inhibitors used are as follows: active caspase 3, poly ADP ribose and vimentin (R&D Systems); Akt, Akt-pS473, ErbB2, ErbB2-pY1248, ErK-pT202/T204, FAK-pY925 and Src-pY416 (Cell Signaling); FAK-pY397, FAK-pY576, FAK-pY577 and paxillin-pY31 (Invitrogen); E-cadherin and paxillin (BD Transduction Laboratories); anti-V5 (Serotec); FAK and N-cadherin (Santa Cruz); actin and tubulin (Sigma); FAK pY407, FAK pY861 and Paxillin pY118 (Biosource); secondary antibodies (Jackson Laboratory); Hoechst 33528 (Sigma). The FAK inhibitor AZ675 was provided by AstraZeneca (Alderly Edge).

### Lentiviral expression

The coding sequences for myrFAK, myrFAK-Y397F, myrFAK-I936/998E, myrFAK-Y925F and v-ErbB2 were cloned in-frame into the pCDH-EF1-MCS-T2A-copGFP lentiviral transfer vector (System Biosciences). The myrFAK sequences have been rpevisouly described [16]; v-ErbB2 was kind gift from Nancy Hynes (Friedrich Miescher Institute, Basel). Stably expressing MCF10A lines were generated by lentiviral infection. HEK293T cells were co-transfected with pPsPax2, pMD2G, and the relevant pCDH transfer vector, and virus production was induced with 10 mM sodium butyrate. Virus was collected after 24-48 hr later, sterile filtered, and added to MECs in 10 μg/ml Polybrene (Millipore). Cells were passaged for 2 weeks and stably expressing cells selected by fluorescence-activated cell sorting (FACS).

### 3-dimensional culture assays

Three-dimensional overlay growth assays were performed as previously described [17], with minor modifications. Briefly, individual 15mm round glass coverslips were evenly coated with Matrigel (BD transduction) to a final thickness of 1mm. Once solidified, a single cell suspension of MCF10A cells in assay media (DMEM/F12 containing 1.8% Horse Serum, 10 ug/ml insulin, 0.5 ug/ml hydrocortisone, 5 ng/ml EGF, 100 ng/ml cholera toxin, 100 U/ml penicillin, and 100 ug/ml streptomycin) supplemented with 2% Matrigel, were seeded atop the solidified Matrigel layer. 4T1 breast cancer MECs assay media: RPMI media containing 10% FBS, 100 U/ml penicillin, and 100 ug/ml streptomycin. Cells were subsequently grown at 37°C, 5% CO_2_ for indicated lengths of time and cultures were overlaid with assay medium supplemented with 2% Matrigel every 4 days. Unstained acinar structures were visualised, at indicated times, using an Olympus Ix51 inverted brightfield/fluorescent microscope and analySIS software (Olympus). Images were subsequently quantified using Image J analysis software.

### Suspension and soft agar culture assays

MCF10A were seeded onto dishes coated with poly-HEMA (Sigma), for indicated lengths of time. Detached cells were subsequently cytospun directly onto poly-lysine slides using a cytospin centrifuge (Shandon), fixed and immunostained as described.

Single-cell suspensions containing 10,000 4T1 breast cancer cells were plated under non-adherent conditions (poly-HEMA-coated) in 35 mm diameter dishes. Spheroids were subsequently drug treated as indicated and spheroid formation subsequently assessed and imaged 72 hours post plating using an Olympus Ix51 inverterted brightfield/fluorescent microscope and analySIS software (Olympus). Images were quantified using Image J analysis software.

For determination of anchorage-independent growth, single-cell suspensions containing 10,000 MCF10A cells in 1.5 ml MCF10a media supplemented with 0.35% low melting point agarose (Sigma) were layered over a base prepared in 35 mm diameter dishes of MCF10a Media and 0.5% agarose. The dishes were incubated for 7 weeks at 37°C in a humidified CO_2_ incubator. Live colonies were stained for 16h at 37°C with nitroblue tetrazolium chloride (1 mg/ml), visualized under a microscope and counted.

### Cell migration assays

To analyse single cell migration, MCF10A cells were plated at a density of 30,000 cells per well, in twelve-well plates and left to adhere and grow for 24 hours. Images were captured every 15 minutes for an additional 24 hours using a Leica AS-MDW inverted microscope and Image Pro 6.3 software (Mediacy). Images were analyses using FIJI Chemotaxis Image analysis software.

For the wound closure assay, confluent MCF10A cells were wounded with a single scratch. To remove cellular debris, wounded monolayers were washed twice in PBS and incubated for 24 hours in complete growth media, with Images being captured every 10 minutes using a Leica AS-MDW inverted microscope and Image Pro 6.3 software (Mediacy). Images were subsequently quantified using FIJI analysis software.

### Protein analysis

Proteins were extracted using 1x RIPA buffer (750 mM NaCl, 250 mM Tris pH 7.4, 25 mM EDTA, 5% NP40, 5% DOC, 0.5% SDS and fresh protease/phosphatase inhibitors; pH 7.5) Equal amounts of proteins were used and equivalent loading assessed by referral to controls, such as tubulin and actin (Sigma). Antibodies were detected using horseradish peroxidase secondary antibodies (Jackson ImmunoResearch) with SuperSignal West Pico Chemiluminescent substrate (Pierce) and Biomax Light film (Kodak).

### Immunofluorescence staining

In 2D cultures, cells were immunostained in PBS, 0.2% Triton X-100, 0.05% Tween 20, 1% horse serum, at room temperature for one hour. Nuclei were stained with Hoechst 33528, and mounted with Prolong® Antifade reagent.

For 3D cultures, coverslips were gently washed with PBS and fixed in 4% paraformaldehyde at room temperature for 30 minutes, permeabilised with 0.5% Triton X-100 in PBS for 5 minutes and subsequently washed three times in IF wash buffer (sterile filtered PBS, 0.1% BSA, 0.2% Triton X-100 and 0.05% Tween-20). Cells were incubated in blocking solution (IF Buffer supplemented with 10% goat serum) and subsequently immunostained with primary antibody diluted in IF Buffer supplemented with 5% goat serum overnight at 4°C. Once washed, cells were incubated with the appropriate fluorescent-tagged secondary anitbody and incubated as before. Coverslips were washed, counterstained with Hoechst 33342 and mounted with Prolong® Gold Antifade reagent.

All fluorescent images were captured with a Leica TCS SP5 AOBS inverted confocal microscope, using a 63× Plan Fluotar objective, and Leica Application Suite software. Image analysis was performed using FIJI analysis software.

### Proliferation assays

For 2D EdU proliferation assays: MCF10a cells were plated into 96 well BD BioCoatTM plate (BD Biosciences) at a density of 30,000 cells per well. Cells treated with either Vehicle (DMSO) or AZ675, were administered 24 hours post-plating and incubated for a further 24 hours prior to cell fixation. One hour prior to fixation with 4% paraformaldehyde at indicated time points, cells were treated with 1uM EdU for 1 hour at 37°C. Once fixed, cells were permeabilised using 0.2% Triton X-100 for 5 minutes. Incorporation of EdU was detected by incubating fixed cells with Alexa fluor 647 for 30 minutes under Cu(I)-catalysed click reaction conditions, according to manufacturer's instructions (Invitrogen). Nuclei were stained with Hoechst 33528 (1:10,000). Cells were visualised using the BD Pathway 855 high throughput bio-imager. Images were obtained and analysed using BD pathway software and FIJI analysis software.

For 3D EdU proliferation assays: MCF10a cells were plated as a single cell suspension on Matrigel mounted coverslips and cultures at 37°C and 5% CO2 for either 7, 12 or 20 days. To quantify cell proliferation, cultures were treated with 1uM EdU, 1 hour prior to fixation and incubated at 37°C and 5% CO2. Following EdU administration, cells were fixed in 4% paraformaldehyde for 30 minutes at room temperature and antibody immunostained as previously described. Following secondary antibody immunostaining and washing, incorporation of EdU was detected by incubating fixed acinar structures with Alexa fluor 647 for 30 minutes under Cu(I)-catalysed click reaction conditions, according to manufacturer's instructions (Invitrogen). Nuclei were stained with Hoechst 33528 (1:10,000). Positive EdU staining was visualised via equatorial acinar confocal cross sectioning using a Leica TCS SP5 AOBS inverted confocal microscope and Leica Application Suite software (Leica). Subsequent image analysis was performed using Fiji image analysis software.

### Conditional knockout mice

The FAKfx/fx and Blg-CreTg/· mice, and morphological and histological analysis have been previously described [22].

## SUPPLEMENTARY FIGURES



## Abbreviations

2D: two dimensional
3D: three dimensional
BLG: β-lactoglobulin
DCIS: ductal carcinoma in situ
ECM: extracellular matrix
EdU: 5-ethynyl-2'-deoxyuridine
EMT: epithelial to mesenchymal transition
FAK: focal adhesion kinase
GFP: green fluorescent protein
MEC: mammary epithelial cells
MMTV: mouse mammary tumour virus
tGFP: turbo GFP
PARP: poly-ADP ribose polymerase
poly-MEMA: Polyhydroxyethyl methacrylate
PyMT: polyoma virus middle T oncoprotein
RIPA: radioimmunoprecipitation assay
SDS-PAGE: SDS polyacrylamide gel electrophoresis
WT: wildtype
