# Supplementary material for: Oncogenic activation of FAK drives apoptosis suppression in a 3D-culture model of breast cancer initiation
Source: Oncotarget. 2016 Sep 6;7(43):70336–52. doi: 10.18632/oncotarget.11856 (PMC5342556; doi:10.18632/oncotarget.11856)
Supplement: Supplementary file 1 [file oncotarget-07-70336-s001.pdf]

Oncogenic activation of FAK drives apoptosis suppression in a 3D-culture model of breast cancer initiation

SUPPLEMENTARY FIGURES

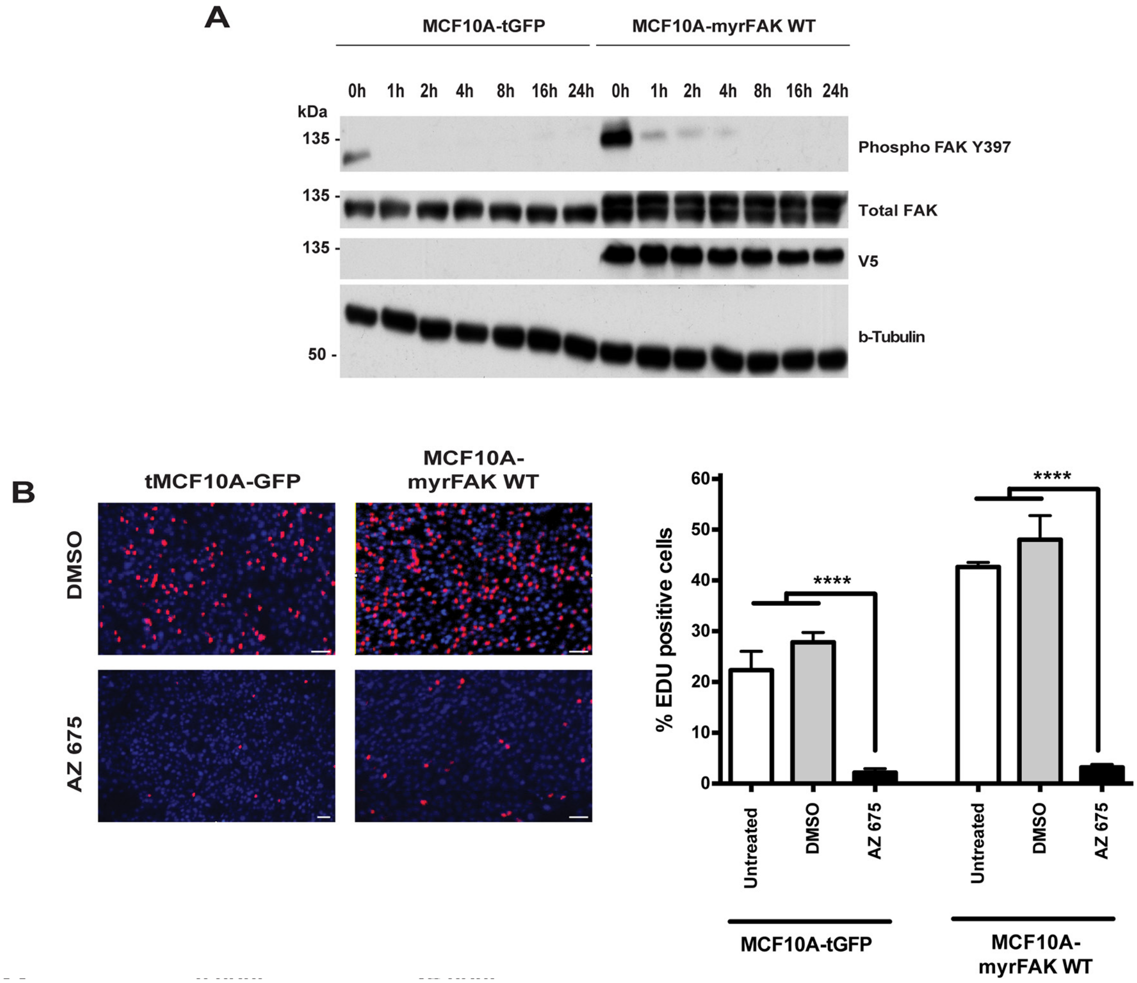

Supplementary Figure S1: Endogenous FAK is required for MCF10A proliferation and migration in 2D-culture. A. MCF10A stably expressing either tGFP or myrFAK were grown in 2D-culture and treated with 5µM AZ675 for the indicated times. Cells were lysed and immunoblotted for phospho-FAK Tyr 397, total FAK, anti-V5 and tubulin. **B.** MCF10A cells expressing tGFP or myrFAK were plated in 2D culture for 24 hours, then treated with either DMSO or AZ675 for a further 24 hours before labelling with Edu for 1 hour before fixation. Edu was detected with Click-iT and nuclei stained with Hoechst. Data show the mean percentage of EdU positive cells from three independent experiments. Error bars = SEM. Data were analysed by ANOVA. **** = $p < 0.0001$. Scale bar = 25 µM. (*Continued*)

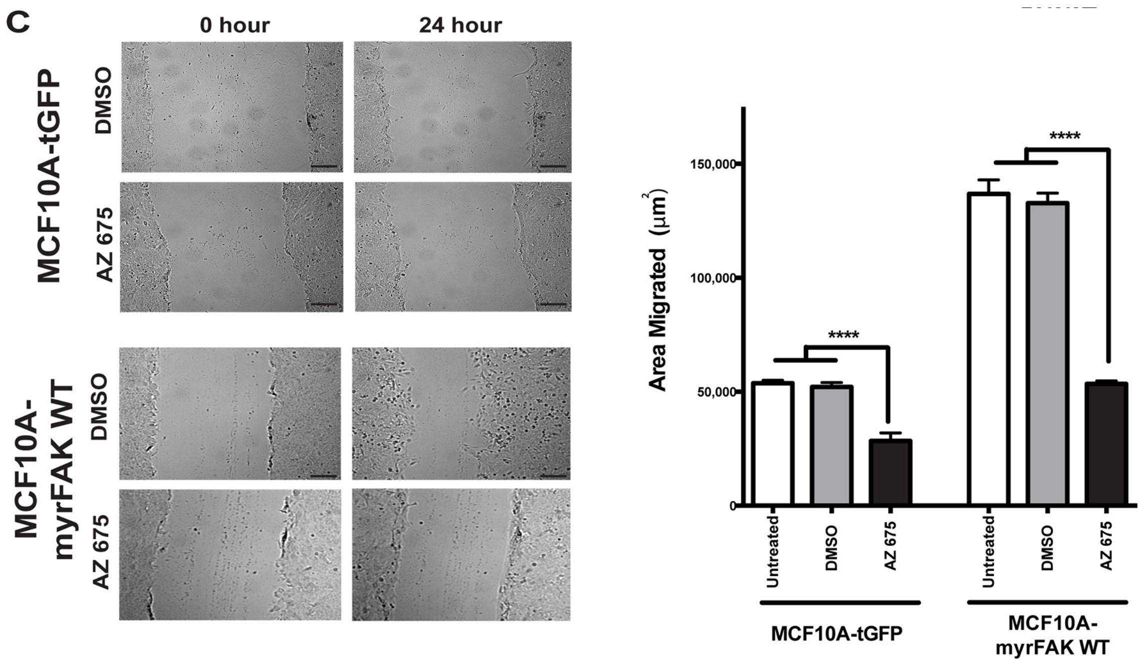

Supplementary Figure S1: (*Continued*) Endogenous FAK is required for MCF10A proliferation and migration in 2D-culture. C. MCF10A cells expressing tGFP or myrFAK were plated in 2D culture and allowed to reach confluence, before wounding with a single scratch. Cells were washed and incubated with DMSO or AZ675 for 24 hours, with images captured every 10 minutes. Representative images are shown from the 24 hours time point. Wound closure was quantified as the wound area occupied by cells after 24 hours. Scale bar = 150 μm. The data represent 15 fields of view from each of three independent experiments. Error bars = SEM. Significance was determined ANOVA. **** = $p < 0.0001$.

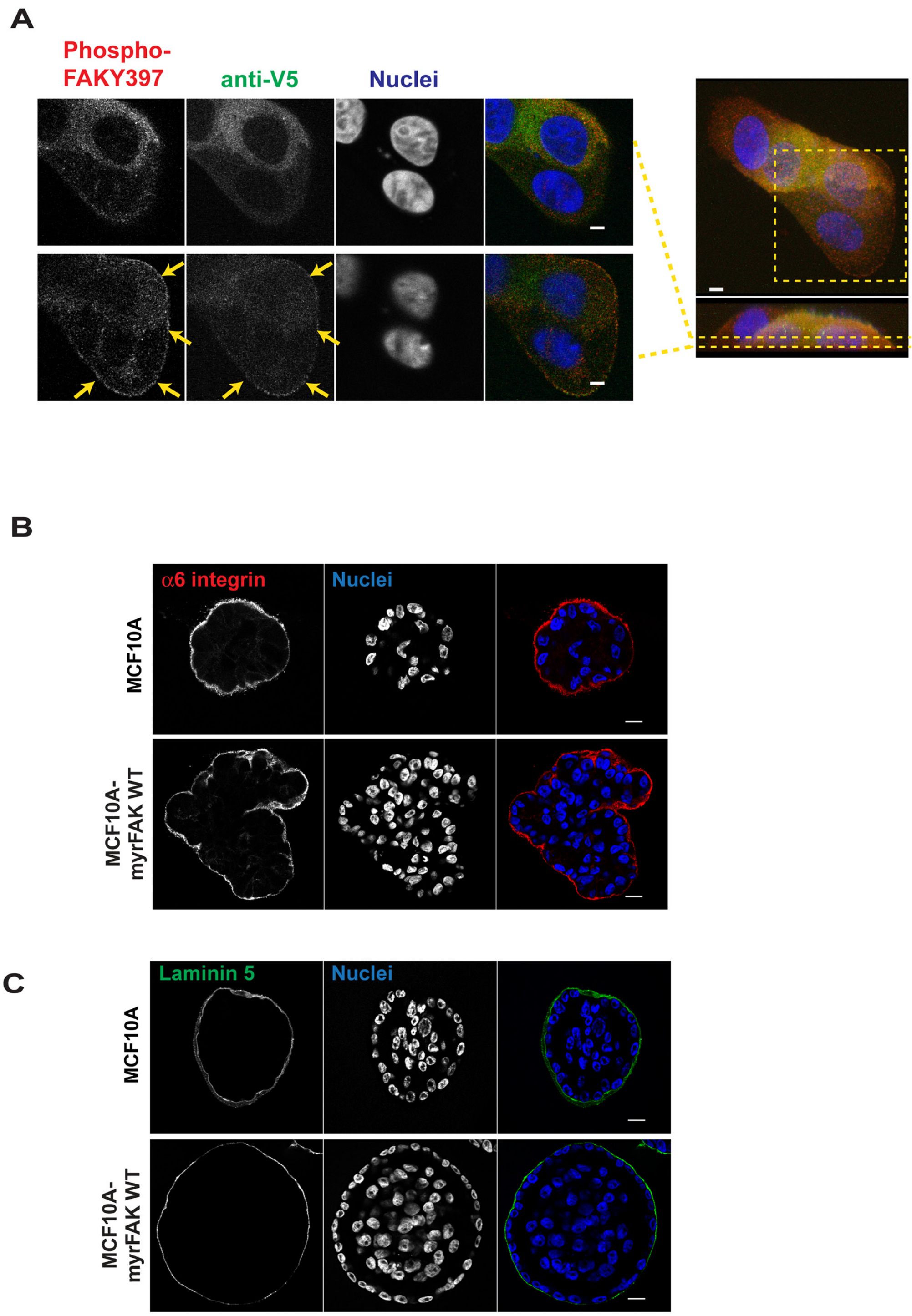

Supplementary Figure S2: myrFAK is expressed throughout MCF10A cells, and does not disrupt basal polarity in MCF10A 3D-cultures. A. MCF10A-myrFAK cells in 2D were fixed and immunostained for V5 and phospho-FAK Tyrosine 397 (Y397). Nuclei were stained with Hoechst. Confocal slices were taken with a z-interval of 1μm, and a 3D-projection is shown in the panel on the right. Two confocal slices (indicated by the dashed lines) are shown for the basal surface in contact with the ECM, and through the nuclei. The basal surface shows clustering of V5 and phospho-Y397 at Focal Adhesions around the cell periphery (indicate by the arrows). Both V5 and phospho-Y397 are also seen throughout the cytosol, but are excluded from the nucleus. **B.** Parental or myrFAK expressing MCF10A cells were grown in 3D matrigel for 10 days, before fixing and immunostaining for integrin α6. myrFAK expression did not disrupt the formation of a basement membrane with associated integrins restricted to this region. Scale bar = 10 μM. **C.** Cells in B. were immunostained for Laminin 5 (LM5). A clear basement membrane is seen around the Day 10 acini of both parental MCF10A cells, and MCF10A-myrFAK cells.

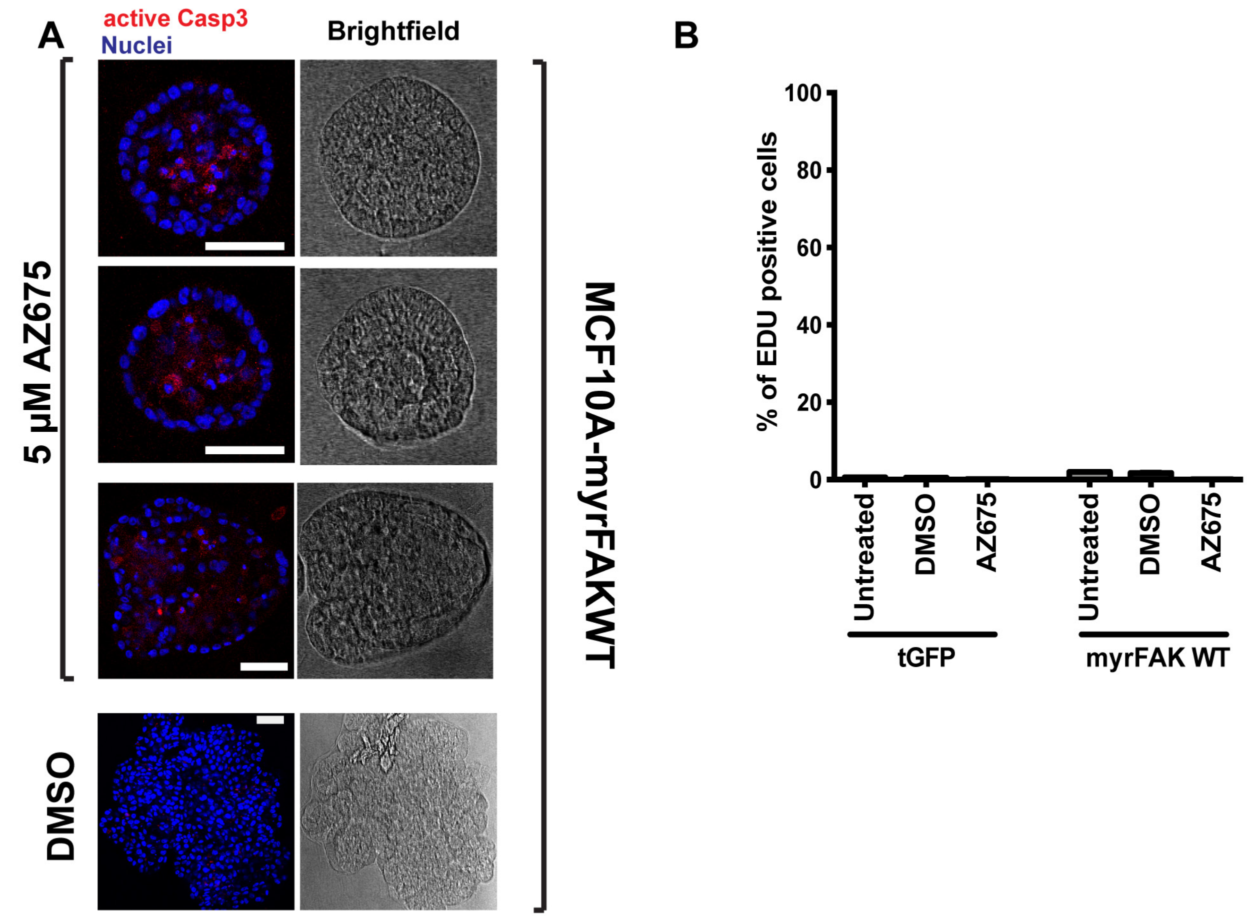

Supplementary Figure S3: Inhibition of myrFAK in 3D-culture selectively induces apoptosis in luminal cells, but not those on the periphery of the acini. A. MCF10A cells stably expressing myrFAK-WT were seeded as single cells in matrigel and cultured for a total of 16 days. The culture media was then replenished with fresh media containing either DMSO or 5 µM AZ675 for a further 96 hours before fixing and immunostaining for active caspase 3. Equatorial confocal sections were taken, along with brightfield images of the acini. Scale bar = 25 µM. Data show that as acini hollow out, apoptosis is restricted to the luminal cells and acinar integrity is maintained. **B.** MCF10A cells stably expressing tGFP, or myrFAK were cultured in matrigel for to 16 days. The culture media was then replenished with fresh media containing either DMSO or 5 µM AZ675 for a further 96 hours. Cells were pulse labelled for one hour with EdU prior to fixation. Cells were immunostained for EdU, and positive cells quantified from three independent experiments. Error bar are SEM. Data were analysed by ANOVA. There is no significant difference in proliferation under any of these conditions.

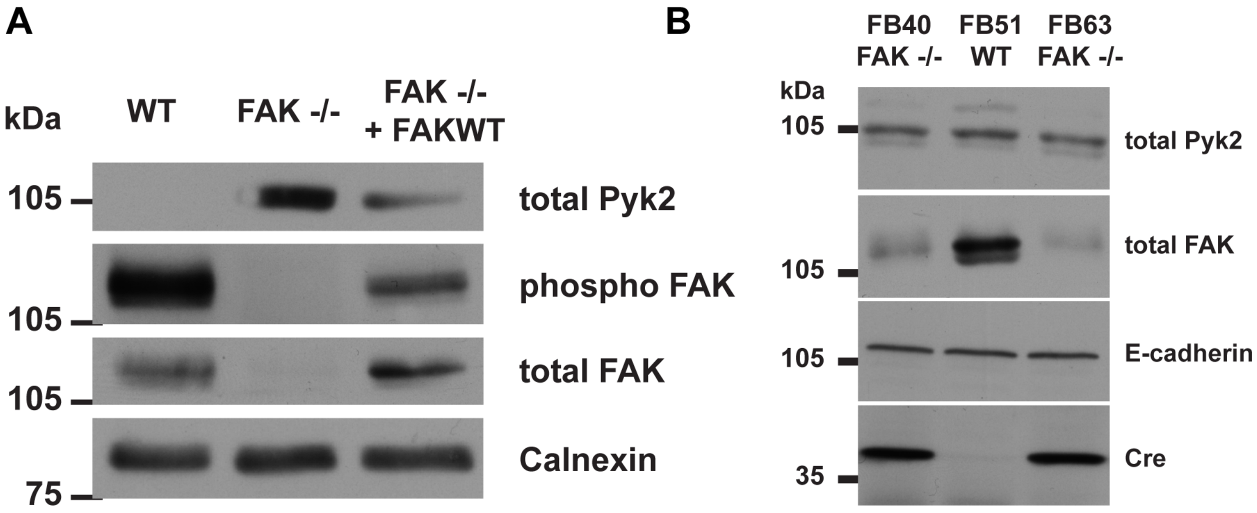

Supplementary Figure S4: Inhibition of FAK in the mouse mammary gland does not result in a compensatory up regulation of Pyk2 expression. A. Loss of FAK expression in mouse embryonic fibroblasts results in Pyk2 up regulation. WT MEFs, Fak -/- MEFs and FAK -/- MEFs stably re-expressing FAK WT, were immunoblotted with the indicated antibodies. Note that as previously described, Pyk2 expression is not seen in WT MEFs, but is present in FAK -/- cells. Re-expression of FAK results in a down regulation of Pyk2 expression. **B.** Whole lysates of mammary glands from one wildtype (WT) and two *FAK fx/fx;BLG-Cre Tg* (*FAK* -/-) mice immunoblotted for the indicated antibodies. All mice had undergone one round of pregnancy, lactation and involution. Note that similar levels of Pyk2 expression are seen in all three.
